# Harnessing reaction-based probes to preferentially target pancreatic β-cells and β-like cells

**DOI:** 10.26508/lsa.202000840

**Published:** 2021-01-28

**Authors:** Sevim Kahraman, Debasish Manna, Ercument Dirice, Basudeb Maji, Jonnell Small, Bridget K Wagner, Amit Choudhary, Rohit N Kulkarni

**Affiliations:** 1 Islet Cell and Regenerative Biology, Joslin Diabetes Center, Department of Medicine, Brigham and Women’s Hospital, Harvard Stem Cell Institute, Harvard Medical School, Boston, MA, USA; 2 Chemical Biology and Therapeutics Science Program, Broad Institute of MIT and Harvard, Cambridge, MA, USA; 3 Department of Medicine, Harvard Medical School, Boston, MA, USA; 4 Divisions of Renal Medicine and Engineering, Brigham and Women’s Hospital, Boston, MA, USA; 5 Chemical Biology Program, Harvard University, Cambridge, MA, USA

## Abstract

Highly sensitive approaches to target insulin-expressing cells would allow more effective imaging, sorting, and analysis of pancreatic β-cells. Here, we introduce the use of a reaction-based probe, diacetylated Zinpyr1 (DA-ZP1), to image pancreatic β-cells and β-like cells derived from human pluripotent stem cells. We harness the high intracellular zinc concentration of β-cells to induce a fluorescence signal in cells after administration of DA-ZP1. Given its specificity and rapid uptake by cells, we used DA-ZP1 to purify live stem cell-derived β-like cells as confirmed by immunostaining analysis. We tested the ability of DA-ZP1 to image transplanted human islet grafts and endogenous mouse pancreatic islets in vivo after its systemic administration into mice. Thus, DA-ZP1 enables purification of insulin-secreting β-like cells for downstream applications, such as functional studies, gene-expression, and cell–cell interaction analyses and can be used to label engrafted human islets and endogenous mouse islets in vivo.

## Abstract (precis)

The study uses a reaction probe to target insulin-expressing pancreatic β-cells and β-like cells derived from pluripotent stem cells by harnessing high intracellular Zn(II) concentration.

## Introduction

Restoring normoglycemia independent of exogenous insulin injections can be achieved by islet cell replacement therapy. However, the limited number of donors and the need for lifelong immunosuppression pose a continuing challenge to the success of islet transplantation approach (Gamble et al, 2018). Human pluripotent stem cells (hPSCs), which have unlimited proliferation potential, provide an excellent source for cell replacement therapies. With recent successes in generating functional pancreatic β-like cells derived from hPSCs, the use of stem cells for the treatment of diabetes is promising (Kahraman et al, 2016). Several laboratories have published protocols to generate functional pancreatic β-like cells in vitro (Pagliuca et al, 2014; Rezania et al, 2014; Russ et al, 2015), and follow-up studies have optimized the protocols to improve the number and function of β-like cells (Zhu et al, 2016a; Ghazizadeh et al, 2017; Nair et al, 2019; Velazco-Cruz et al, 2019; Hogrebe et al, 2020). However, several limitations have emerged with the directed differentiation of hPSCs for research and therapy. First, current protocols for making hPSC-derived β-like cells result in cell cultures that consist of a mixture of cell types, including non-β endocrine cells and cells with tumorigenic potential, which could develop into tumors after transplantation (Nair et al, 2019; Veres et al, 2019). The second concern is the known variability across hPSC lines, which results in generation of variable numbers of insulin-expressing cells at the final stage of the differentiation protocol (Thatava et al, 2013). To circumvent these issues, several groups have developed methods to isolate and purify insulin-secreting β-like cells for transcriptional and functional analyses. One such approach uses *INS*^GFP/w^ human embryonic stem cells (hESCs) to facilitate isolation of insulin-expressing cells (Micallef et al, 2012). However, this approach depends on generating a reporter line and is not convenient when using other unmodified hPSC or patient-derived induced pluripotent stem cell (iPSC) lines (Zhu et al, 2016b). Other approaches include cellular fixation followed by intracellular immunofluorescence and sorting (Hrvatin et al, 2014); although this is useful for transcriptional analyses, it cannot be used for downstream functional studies requiring live cells. Other groups have reported using cell surface antibodies to alternately sort pancreatic progenitors or endoderm cells to increase the yield of β-like cells during the terminal differentiation stages (Kelly et al, 2011; Ameri et al, 2017; Cogger et al, 2017). Although this method allows elimination of undifferentiated human embryonic stem cells, further differentiation of sorted pancreatic progenitors into β-like cells results in generation of C-peptide+ (CPEP) cells at varying efficiencies. Recently, magnetic cell sorting of β-like cells using the cell surface marker, CD49a, has been reported to enrich β-like cells derived from hPSCs; however, this method requires antibody staining (Veres et al, 2019).

A small-molecule–based method for sorting has several advantages over the aforementioned genetic- and biologic-based methods. First, unlike biologics, small molecules can be easily delivered into cells through passive diffusion allowing targeting of cell-specific markers located intracellularly or on cell surface without cellular fixation, reducing both effort and cost, and allowing scaling up of cell sorting processes. Second, unlike genetic methods, small molecules act rapidly requiring as little as a few minutes for their activity (Que et al, 2008). Such rapid kinetics allows precision dose- and temporal-control and enables fine-tuning of the activity and specificity of the sorting method. Third, small molecules are typically not immunogenic and thereby enable in vivo applications. Finally, small molecules have the advantage of being able to be produced *en masse* and at low cost with little batch-to-batch variability. Prior efforts have used the zinc content in β-cells to sort human and murine pancreatic β-cells, using fluorescent probes such as TSQ (6-methoxy-8-p-toluenesulfonamido-quinoline) (Klochendler et al, 2016), FluoZin-3-AM (Jayaraman, 2008), or Newport Green (Parnaud et al, 2008). TSQ has also been used for purification of β-like cells derived from hPSCs based on intracellular zinc content (Davis et al, 2019). Here, we employed a reaction-based probe, diacetylated Zinpyr1 (DA-ZP1), to label and isolate insulin-expressing cells both in vivo and in vitro. DA-ZP1 is non-fluorescent in the absence of zinc ions [Zn(II)], but binding of Zn(II) selectively and rapidly mediates hydrolytic cleavage of the acetyl groups, providing a detectable fluorescence response (Chyan et al, 2014). Lippard and co-workers (Zastrow et al, 2016) demonstrated the Zn(II) specificity of this reaction over those of other biologically relevant metal ions, including Fe(II), Cu(II), Mn(II), Co(II), and Ni(II). Insulin-secreting cells are selectively enriched for Zn(II), whereas α cells and pancreatic exocrine cells exhibit relatively less abundance of the ion (Toroptsev et al, 1974; Jindal et al, 1992). Indeed, insulin-containing cells are highly enriched for Zn(II) to a magnitude of 10–20 mM in insulin granules (Li, 2014), making them an excellent target cell type for zinc-dependent fluorescence labeling (Jindal et al, 1992).

Furthermore, tracking β-cell mass in vivo serves an important role for assessing outcomes of therapeutic interventions for diabetes. In this context, high β-cell specificity relative to neighboring endocrine and exocrine cells is an essential parameter for successful in vivo imaging of β-cell mass (Sweet et al, 2004). Considering Zn(II) is considerably enriched in pancreatic β-cells compared with neighboring cells and no toxicity is observed in in vivo studies (Rice et al, 2016), zinc-based reaction probes are promising candidates for β-cell imaging. In efforts to label pancreatic β-cells in vivo, we also tested a chelator-based strategy to monitor engrafted human islets and endogenous mouse islets. Our work shows that systemic administration of DA-ZP1 leads to its enrichment in pancreatic β-cells and has the potential to be developed for measuring β-cell mass, sorting, and targeting.

In this study we report the in vitro use of zinc- and small-molecule–based reaction probes to label and image live insulin-secreting β-like cells in real time. Our data indicate that DA-ZP1 preferentially labels insulin-positive cells and can be used to enrich live insulin-positive cells for multiple downstream applications, including gene expression analysis, functional studies, cell–cell interaction analyses, in vivo imaging, and β-cell directed activation of bioactive small molecules.

## Results

### DA-ZP1 is preferentially unmasked in pancreatic β-cells in a time- and dose-dependent manner without affecting function

**Figure 1.**
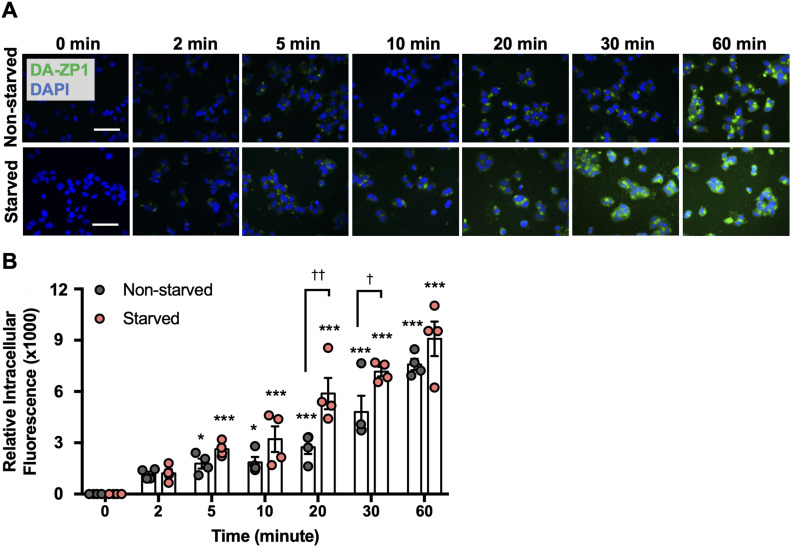
Time course accumulation of DA-ZP1 in human pancreatic β-cells. **(A)** Starved (3-h starvation in DMEM containing 2 mM glucose) or non-starved EndoC-βH1 cells were treated with 5 μM DA-ZP1 for 0, 2, 5, 10, 20, 30, or 60 min and then fixed in 4% paraformaldehyde. Representative images show presence of DA-ZP1 (green) in EndoC-βH1 cells at the different times. Nuclei stained with DAPI (blue). Scale bar is 500 μm. **(B)** Quantification of intracellular fluorescence 0, 2, 5, 10, 20, 30, or 60 min after DA-ZP1 treatment. Relative intracellular fluorescence was measured using Image J. Staining was conducted in a 96-well format (n = 4 replicates/condition), and at least four images per condition were captured per well. Data are represented as mean ± SEM. *P < 0.05, ***P < 0.001 versus 0 min, †*P* < 0.05, ††*P* < 0.01 starved versus non-starved. Two-way ANOVA followed by the Holm–Sidak method.

**Figure 2.**
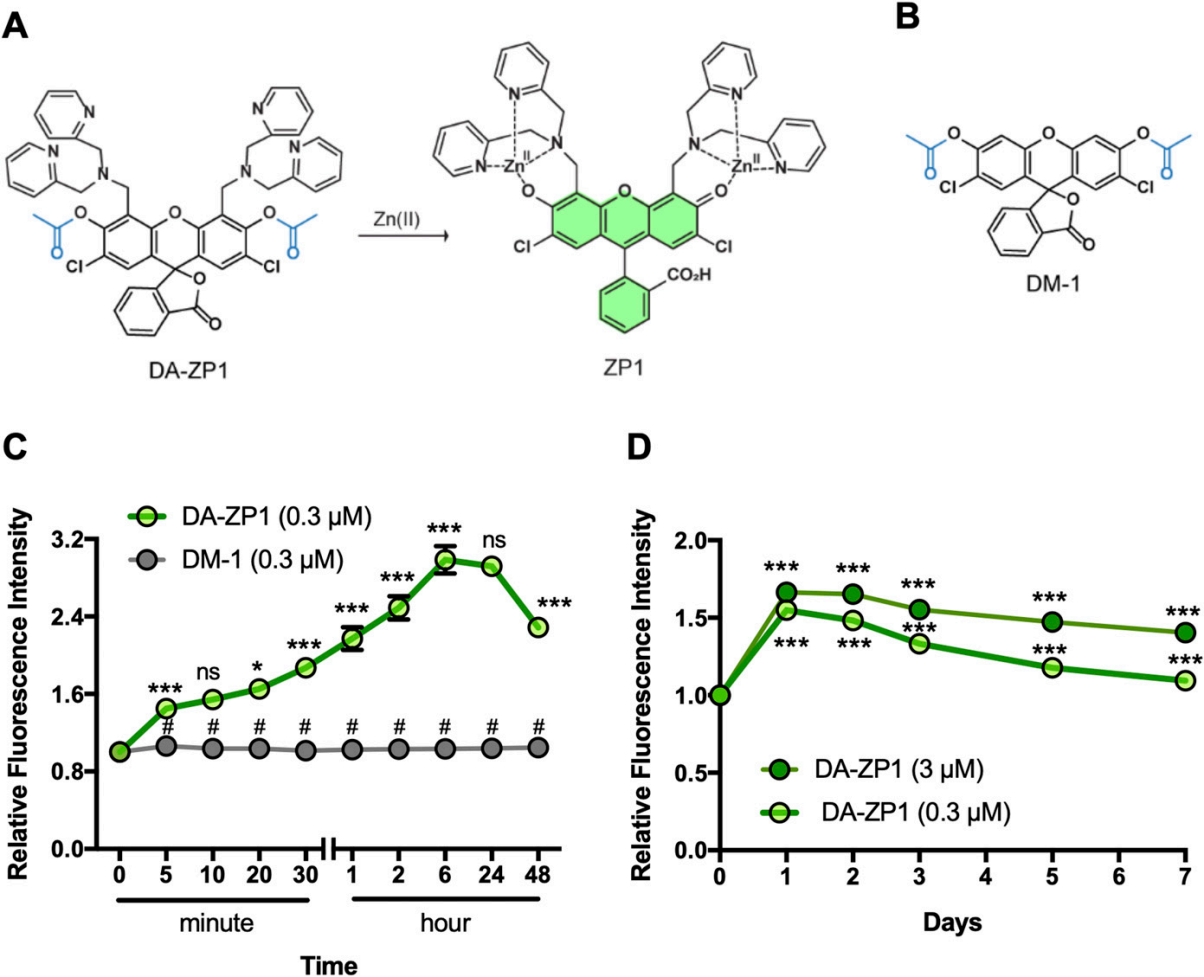
DA-ZP1 and DM-1 staining of human pancreatic β-cells. **(A)** Chemical structure of DA-ZP1. Zn(II)-mediated deacetylation of DA-ZP1 releases active fluorophore ZP-1. **(B)** Chemical structure of control compound DM-1 which is a DA-ZP1 analog lacking the Zn(II)-binding ligand. **(C)** EndoC-βH1 cells were treated with DA-ZP1 (0.3 μM) or DM-1 (0.3 μM) for 0, 5, 10, 20, 30 min, or 1, 2, 6, 24, or 48 h. Staining was conducted in a 96-well format (n = 4 replicates/condition). Fluorescent images were acquired using an automated system (9 images/well) and intracellular fluorescence was measured by MetaXpress software. Relative fluorescence intensity showed presence of DA-ZP1 (green line) and DM-1 (gray line) at different time points in EndoC-βH1 cells. #*P* < 0.0001 DA-ZP1 versus DM-1, ***P < 0.0001 DA-ZP1 5 versus 0 min, 30 versus 20 min, 1 h versus 30 min, 2 versus 1 h, 6 versus 2 h, 48 versus 24 h, *P < 0.05 DA-ZP1 20 versus 10 min, ns indicates not significant 10 versus 5 min, 24 versus 6 h. Two-way ANOVA followed by Tukey’s method. **(D)** Relative fluorescence intensity was measured for 7 d after treatment of EndoC-βH1 cells with DA-ZP1 (0.3 μM; light green, 3 μM; dark green, n = 8 replicates/condition). Data are represented as mean ± SEM. Two-way ANOVA followed by Dunnett’s method. ***P < 0.0001 versus 0 min.

To begin, we tested DA-ZP1 in human pancreatic β-cells for fluorescence imaging over time. EndoC-βH1 cells were treated with 5 μM DA-ZP1 and fluorescence intensity was measured over 1 h (Fig 1A). An increase in the intracellular fluorescence signal was observed in cells 2 min after administration of DA-ZP1 and the signal increased significantly over 1 h (Fig 1B). Starved cells displayed stronger fluorescence signal compared with non-starved cells possibly because starvation induces an enhanced ability to detect Zn(II) in pancreatic β-cells (Toroptsev et al, 1974). To ensure that the unmasking of DA-ZP1 fluorescence in cells was not catalyzed by an esterase and the fluorescence is specific to Zn(II) binding, we synthesized a DA-ZP1 analog (DM-1) lacking the Zn(II)-binding ligand (and contains the esters) (Fig 2A and B) and tested its effects on EndoC-βH1 cells. As expected, EndoC-βH1 cells treated with DM-1 exhibited little to no fluorescence compared with DA-ZP1–treated cells, even up to 48 h after treatment (Fig 2C). DA-ZP1, on the other hand, generated strong fluorescence, which was significantly increased in the first 6 h and then significantly declined at 48 h. Prolonged treatment with DA-ZP1 showed that the fluorescence signal could be detected even after 7 d (Fig 2D). Whereas the half-life of DA-ZP1 for highly active porcine esterase is in the range of hours the same for Zn(II) is ∼8.1 s, indicating a kinetic specificity to the DA-ZP1 scaffold for Zn(II) (Chyan et al, 2014). Furthermore, DA-ZP1 is a PET-based zinc sensor that requires Zn(II)-triggered hydrolysis of a tethered acetate ester to turn-on fluorescence and is insensitive to intracellular esterases (Zastrow et al, 2016).

**Figure 3.**
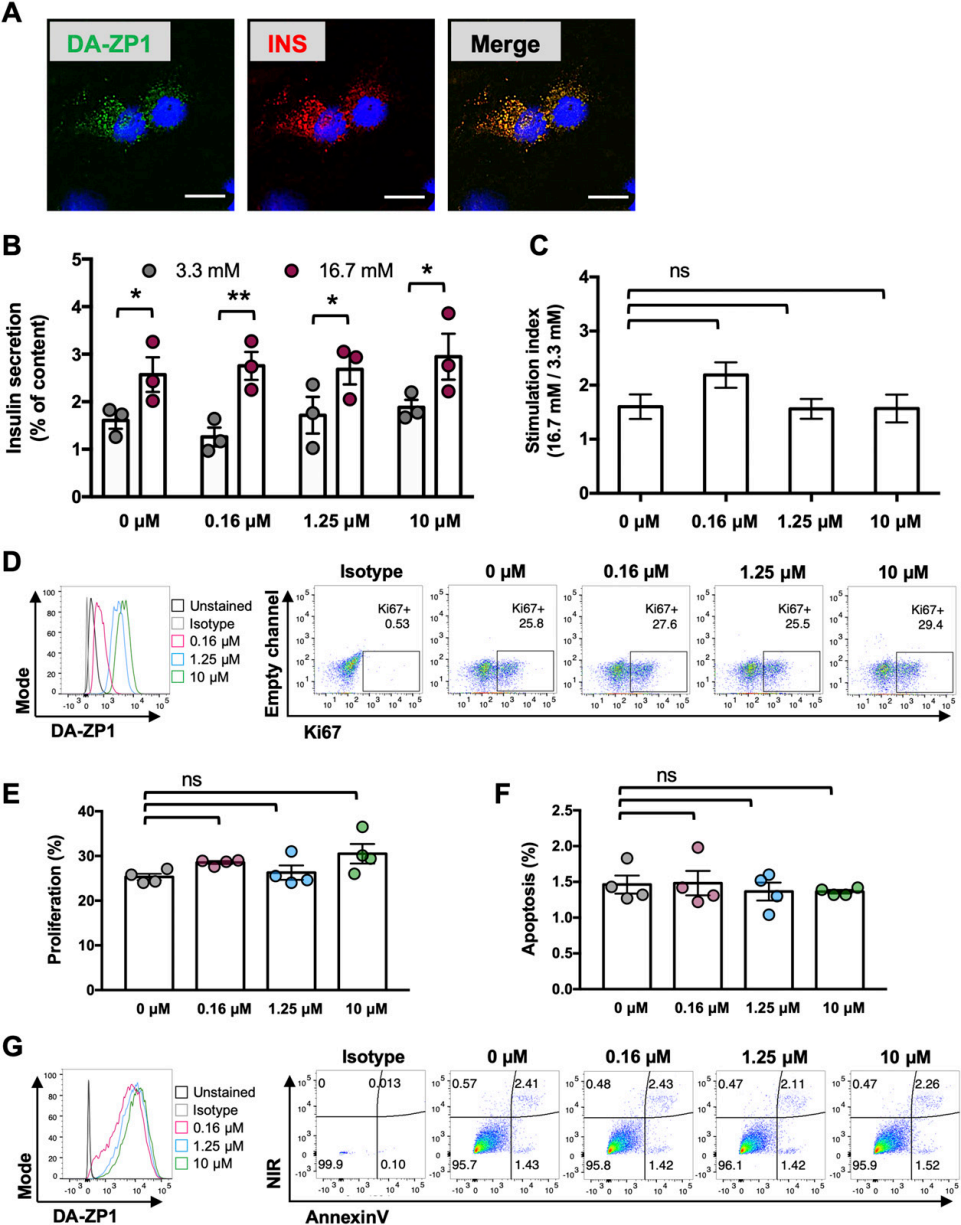
DA-ZP1 treatment does not impair β-cell function. **(A)** Confocal imaging of EndoC-βH1 cells stained with DA-ZP1 (60 min, 5 μM DA-ZP1) and subsequently fixed and co-stained for insulin showing DA-ZP1 staining in subcellular compartments of the cells. Nuclei stained with DAPI (blue). Scale bar is 20 μm. **(B)** Glucose stimulated insulin secretion assay performed after treatment of EndoC-βH1 cells with 0, 0.16, 1.25, or 10 μM DA-ZP1 (n = 3 independent replicates/condition). Insulin secretion plotted as % of total insulin content. Data are represented as mean ± SEM. *P < 0.05, **P < 0.01. *P*-values were calculated by unpaired multiple *t* test to determine differences between 16.7 and 3.3 mM. **(C)** The stimulation index was calculated as the fold increase in insulin release measured in 16.7 over 3.3 mM glucose (n = 3 independent replicates/condition). *P*-values were calculated by one-way ANOVA with Dunnett for multiple comparison versus 0 μM. **(D, E, F, G)** Effects DA-ZP1 treatment on proliferation (D, E) and apoptosis (F, G) in EndoC-βH1 cells (n = 4 replicates/condition). *P*-values were calculated by one-way ANOVA followed by Dunnett’s method. Data are represented as mean ± SEM. ns indicates not significant.

“Mobile” Zn(II) is found in concentrations between 0.4 and 1.5 nM in the cytosol and at substantially higher concentrations (1–100 μM) in insulin-containing secretory vesicles in pancreatic β-cells (Vinkenborg et al, 2009; Rutter et al, 2016). Consistently, we observed presence of the fluorescence signal in subcellular compartments in the EndoC-βH1 cells colocalizing with insulin (Fig 3A). Since DA-ZP1 binds to Zn(II), which are essential for the correct processing, storage, and secretion of insulin (Emdin et al, 1980; Li, 2014; Rutter et al, 2016), it is possible that zinc chelation by DA-ZP1 disrupts normal insulin secretory function. To exclude this possibility, we treated EndoC-βH1 cells with increasing concentrations of DA-ZP1 for 30 min and then measured glucose-stimulated insulin secretion (Fig 3B and C). Both treated and untreated β-cells responded to high glucose (16.7 mM) by stimulating insulin secretion significantly and equally, suggesting that DA-ZP1 does not acutely interfere with β-cell secretory function. To assess other potential negative effects on β-cells, treated cells were cultured for 24 h followed by evaluation of changes in apoptosis and cell proliferation (Fig 3D–G). The lack of significant differences between treated and untreated cells indicated that DA-ZP1 treatment does not affect cellular viability and proliferation.

### Low concentrations of DA-ZP1 differentiate β-cells from non-β cells

**Figure 4.**
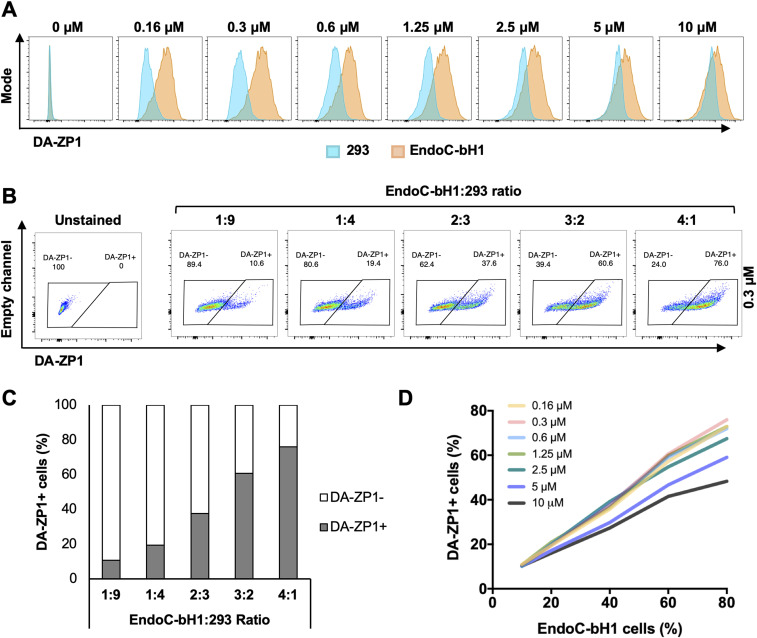
Low concentrations of DA-ZP1 differentiate β-cells from non-β cells. **(A)** EndoC-βH1 and 293 cells were treated with different concentrations of DA-ZP1 for 30 min and then analyzed by flow cytometry (n = 3 replicates for each dose/group). Blue histogram shows 293 cells and orange histogram shows EndoC-βH1 cells. See also Fig S1A. **(B)** EndoC-βH1 cells were mixed with 293 cells in different proportions (EndoC-βH1:293 ratio; 1:9, 1:4, 2:3, 3:2, and 4:1), stained with 0.3 μM DA-ZP1 for 30 min, and analyzed by flow cytometry (n = 5 samples). See also Fig S1B for other concentrations of DA-ZP1. See also Fig S2A and B. **(C)** Quantification of percentage of DA-ZP1+ cells shows correlation with EndoC-βH1 proportion in the cell mixture (n = 5 samples). See also Fig S1C for other concentrations of DA-ZP1. See also Fig S2C. **(D)** Correlation of percentage of DA-ZP1+ cells and percentage of EndoC-βH1 cells in the mixed samples stained with different concentrations of DA-ZP1 (n = 5 samples) (Pearson’s correlation r = 0.99 for each concentration). See also Fig S2D.

**Figure S1.**
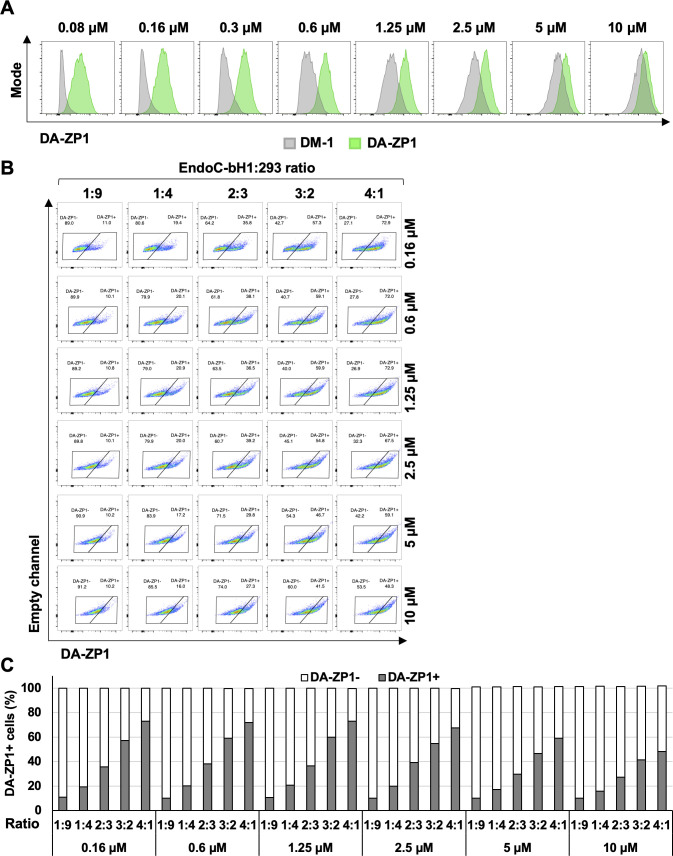
DA-ZP1 differentiates β-cells from non-β cells. **(A)** FACS analysis of DM-1 and DA-ZP1 stained EndoC-βH1 cells. EndoC-βH1 cells were treated with DM-1 and DA-ZP1 at different concentrations (0, 0.08, 0.16, 0.3, 0.6, 1.25, 2.5, 5, or 10 μM) for 30 min and analyzed by flow cytometry (n = 3 replicates for each dose/group). Cell count (y-axis) is normalized to mode. **(B)** EndoC-βH1 cells were mixed with 293 cells in different proportions (EndoC-βH1:293 ratio; 1:9, 1:4, 2:3, 3:2, 4:1), stained with 0.16, 0.6, 1.25, 2.5, 5, or 10 μM DA-ZP1 for 30 min, and % of DA-ZP1− and DA-ZP1+ cells were determined by flow cytometry (n = 5 samples). **(C)** Quantification of percentage of DA-ZP1+ cells correlates with the proportion of EndoC-βH1 in the cell mixture (n = 5 samples).

To test the fluorescent activity of DA-ZP1 by a flow cytometer, we treated EndoC-βH1 and 293 cells with DA-ZP1 for 30 min followed by FACS analyses. Lower concentrations of DA-ZP1 resulted in brighter staining in EndoC-βH1 compared with 293 cells with little overlap (Fig 4A). However, higher doses of DA-ZP1 resulted in comparable and overlapping staining in both cell types. Using the DM-1 control analog, we confirmed that low concentrations of DA-ZP1 are specific to EndoC-βH1 cells, whereas higher concentrations of each compound produce background noise (Fig S1A). Binding of DA-ZP1 with Zn(II) at concentrations substantially greater than the *K*d value of DA-ZP1, which is 0.60 ± 0.03 nM in cuvette studies (Chyan et al, 2014), is one possible explanation for the nonspecific binding and fluorescence release at higher concentrations (≥1.25 μM). Other metal ions, such as Co(II) and Cu(II), could cleave the acetyl groups and also generate fluorescence signals but generally their levels within the cell are not adequate for this reaction. In addition, Zynpyr sensors show selectivity for Zn(II) over other biologically relevant metal ions (Chyan et al, 2014; Zastrow et al, 2016).

**Figure S2.**
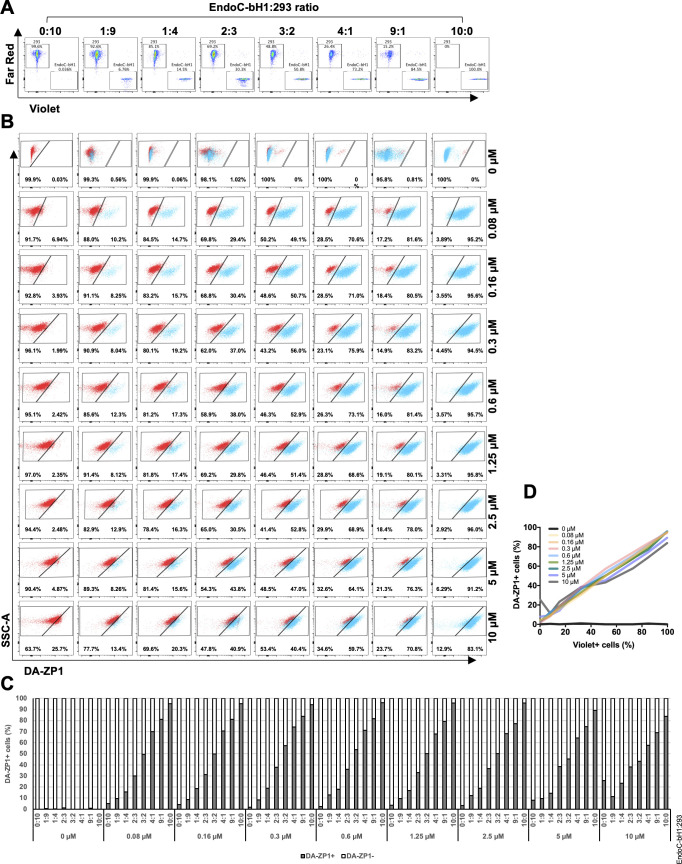
DA-ZP1 is specific to β-cells. **(A)** EndoC-βH1 cells and 293 cells were labeled with cell trace dye Violet and Far Red, respectively. Labeled cells were mixed at different proportions (EndoC-βH1:293 ratio; 0:10, 1:9, 1:4, 2:3, 3:2, 4:1, 9:1, or 10:0) (duplicated samples were analyzed per mixture, n = 8). **(B)** FACS plots showing EndoC-βH1 cells in blue and 293 cells in red. Mixed cells were stained with 0, 0.08, 0.16, 0.6, 0.3, 1.25, 2.5, 5, or 10 μM DA-ZP1 for 30 min, and % of DA-ZP1− and DA-ZP1+ cells were determined by flow cytometry (duplicated samples were analyzed per mixture, n = 8). **(C)** Quantification of percentage of DA-ZP1+ cells shows correlation with percentage of Violet+ cells (EndoC-βH1) in the cell mixture (duplicated samples were analyzed per mixture, n = 8). **(D)** Correlation of percentage of DA-ZP1+ cells and percentage of Violet+ cells (EndoC-βH1) in the mixed samples. Pearson’s correlation r = 0.99 for all except 0 μM (r = 0.05) (duplicated samples were analyzed per mixture, n = 8).

**Figure S3.**
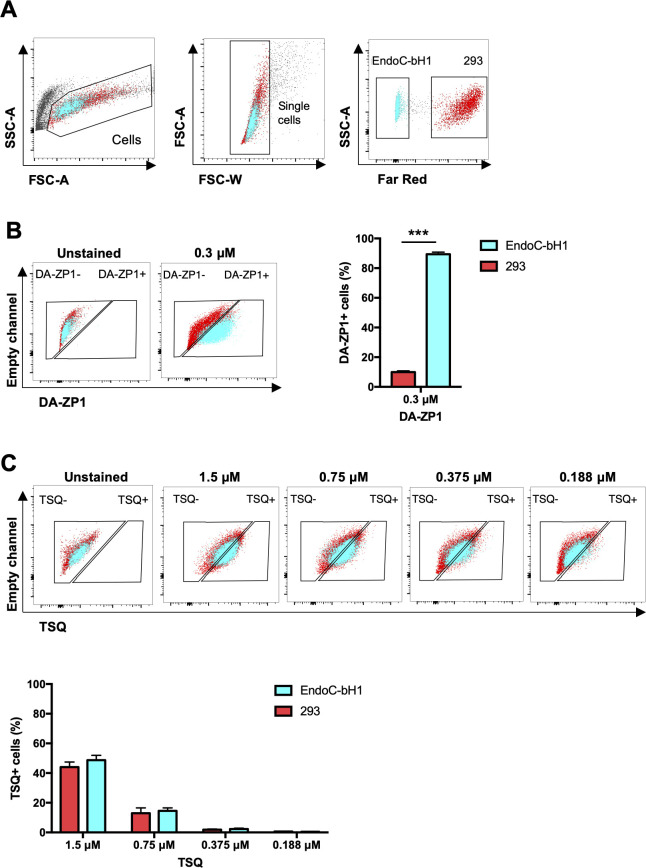
DA-ZP1 discriminates β-cells from non-β cells more effectively than TSQ. **(A)** Representative FACS plot showing gating strategy of mixture of EndoC-βH1 cells (blue) and Far Red labeled 293 cells (red) (1:1 ratio). **(B)** Percentage of β and non-β cells in the DA-ZP1+ fraction. **(C)** Percentage of β and non-β cells in the TSQ+ fraction. n = 3 replicates. Data are represented as mean ± SEM. Multiple *t* test followed by the Holm–Sidak method. ***P < 0.001.

We then sought to determine the ability of DA-ZP1 to sort β from non-β cells. We mixed EndoC-βH1 and 293 cells in different ratios (1:9, 1:4, 2:3, 3:2, and 4:1) and treated the mixture of cells with DA-ZP1 for 30 min (Figs 4B and S1B). The percentage of DA-ZP1+ cells showed a positive correlation with the proportion of EndoC-βH1 cells in the mixed cell population (Pearson’s correlation *r* = 0.99 for each concentration), suggesting that DA-ZP1 is able to sort β-cells from a mixed cell population (Figs 4C and D and S1C). To confirm that DA-ZP1+ cells correspond to the β-cells in the mixed cell population, each cell type was first labeled with a different dye (EndoC-βH1 with Violet and 293 with Far Red) that could be used to track them after mixing the cell types (Fig S2A). Very high positive correlation (*r* = 0.99) (Hinkle et al, 2003) between DA-ZP1+ cells and Violet+ cells confirmed that DA-ZP1 was specific to β-cells (Fig S2B–D). To compare sorting efficiency of DA-ZP1 with other zinc probes such as TSQ, we analyzed a mixture of β and non-β cells (1:1 ratio EndoC-βH1:Far Red-labeled 293) after DA-ZP1 or TSQ staining (Fig S3A). Whereas DA-ZP1 effectively discriminated β-cells from non-β cells, TSQ failed to preferentially label the former at any of the concentrations tested (Fig S3B and C).

### DA-ZP1 enables sorting of live hESC-derived β-like cells

**Figure 5.**
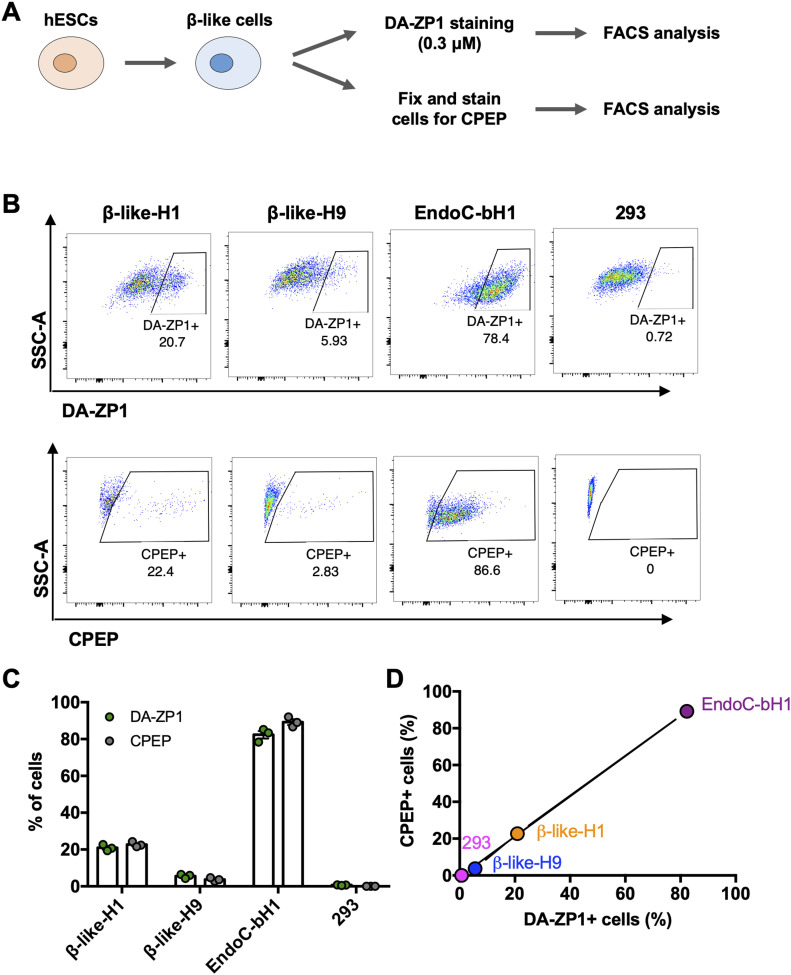
Percentage of DA-ZP1+ cells correlates with percentage CPEP+ cells. **(A)** Human embryonic stem cells were differentiated to β-like cells and first stained for DA-ZP1 (0.3 μM) followed by CPEP staining. Composition of human embryonic stem cell differentiation media is available in Table S1. **(B)** FACS analysis of β-like cells derived from two different human stem cell lines (β-like-H1 and β-like-H9), EndoC-βH1, and 293 cells stained for DA-ZP1 (0.3 μM) or CPEP (n = 3 replicates). See also Fig S4. **(C)** Quantification of FACS data for percentage of DA-ZP1+ cells and CPEP+ cells (n = 3 replicates). Data are mean ± SEM. **(D)** Correlation of percentage of DA-ZP1+ cells and percentage of CPEP+ cells (Pearson’s correlation r = 0.99) (n = 3 replicates). See also Fig S4.

**Figure S4.**
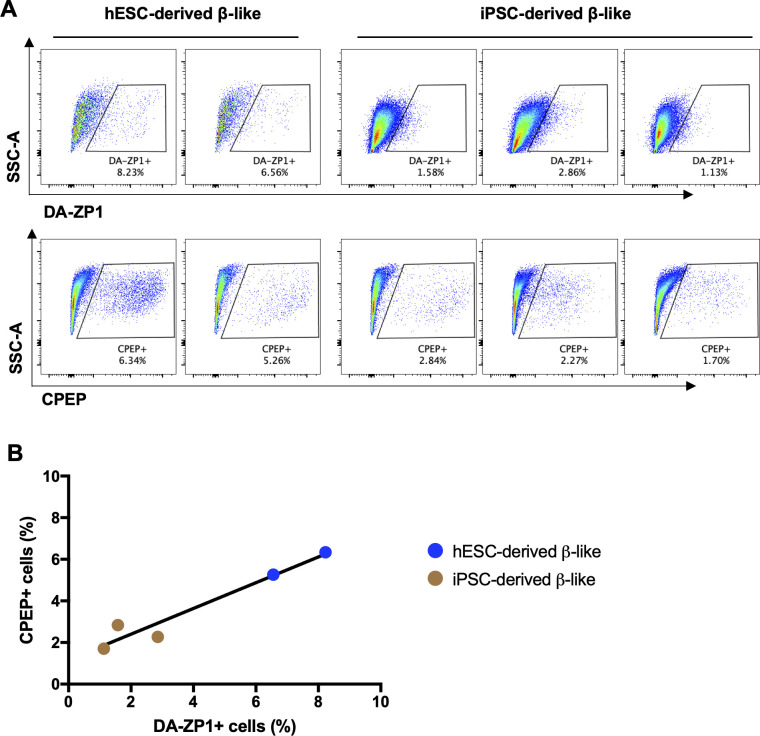
DA-ZP1 stains β-like cells derived from human embryonic stem cell and induced pluripotent stem cells. **(A)** Representative FACS plots showing DA-ZP1 (upper panel) and CPEP (lower panel) stained β-like cells derived from human embryonic stem cell line (H1) and three human induced pluripotent stem cell lines. **(B)** Correlation of percentage of DA-ZP1+ β-like cells and percentage of CPEP+ β-like cells (n = 5 biological replicates).

**Figure 6.**
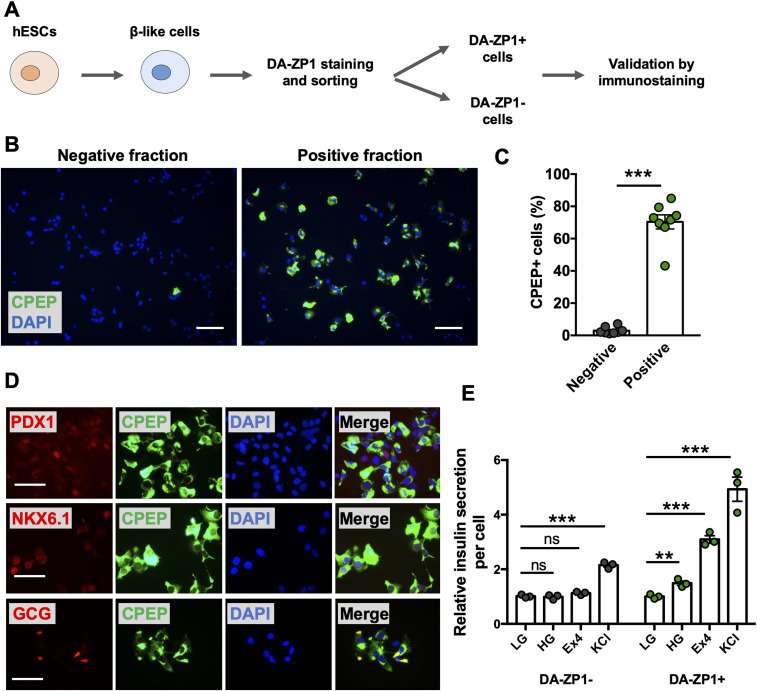
Enrichment of CPEP+ cells in DA-ZP1 positive fractions. **(A)** Overview of DA-ZP1 analysis and experimental approach. **(B)** β-like cells derived from two human embryonic stem cell lines were stained and DA-ZP1+ and DA-ZP1− cells were collected after FACS. Representative images show CPEP staining (green) in DA-ZP1+ and DA-ZP1− fractions. Nuclei stained with DAPI (blue). Scale bar is 100 μm. **(C)** Quantification of percentage of CPEP+ cells in DA-ZP1+ and DA-ZP1− fractions. Staining was conducted in a 96-well format (n = 8, two biological replicates with four technical replicates). At least six images per condition were captured and >1,000 cells were analyzed per well. Data are represented as mean ± SEM. ***P < 0.001, *P*-value is calculated by two-tailed *t* test. **(D)** Immunostaining of DA-ZP1+ cells that were collected after FACS for markers of pancreatic β-cells (n = 3 technical replicates). PDX1 (red), NKX6.1 (red), GCG (red), CPEP (green), and DAPI (blue). Scale bar is 50 μm. **(E)** Static insulin secretion test was performed on DA-ZP1 sorted positive and negative cells at β-like cell stage (n = 3 replicates/condition, repeated twice using two different human embryonic stem cell lines). Human insulin secretion was calculated by dividing the secreted insulin by the total number of cells. The amount of insulin secretion was normalized to the amount of insulin secreted in LG condition. LG, low glucose (1 mM); HG, high glucose (20 mM); Exendin-4 (Ex4), 10 nM Ex4 in the presence of 20 mM glucose; KCl, 30 mM KCl. Data are represented as mean ± SEM. ns indicates not significant. **P < 0.01, ***P < 0.001 versus LG. *P*-values were calculated by multiple *t* test followed by the Holm–Sidak method.

We then sought to determine whether DA-ZP1 could be used to sort β-like cells from differentiating stem cell populations. We differentiated two different hESC lines, H1 and H9, into β-like cells (β-like-H1 and β-like-H9) and treated them with 0.3 μM DA-ZP1 (Fig 5A). EndoC-βH1 and 293 cells served as positive and negative control cell lines, respectively. The percentage of CPEP cells in the same samples was determined by fixing and immunostaining for CPEP (Fig 5B). The β-like cells derived from the two lines displayed some DA-ZP1 staining (β-like-H1, 20.9% ± 1.66% DA-ZP1+; β-like-H9, 5.57% ± 1.13% DA-ZP1+). Whereas differentiation of the two hESC lines into β-like cells (β-like-H1 and β-like-H9) produced different yields of CPEP+ cells (β-like-H1, 22.73% ± 1.43% CPEP+; β-like-H9, 3.70% ± 0.92% CPEP+), the percentage of CPEP+ cells correlated with the percentage of DA-ZP1+ cells (Pearson’s correlation *r* = 0.99) (Fig 5C and D). We used the same approach to test β-like cells derived from human iPSCs; again comparable staining of DA-ZP1 and CPEP was observed in β-like cells derived from three iPSC cell lines (Fig S4A and B). To confirm preferential accumulation of DA-ZP1 in CPEP+ β-like cells, we collected DA-ZP1 positive and negative cells after FACS and analyzed them for their hormone content by immunostaining (Fig 6A). CPEP+ β-like cells were enriched by 23.6-fold in the positive fraction compared with negative fraction (Fig 6B and C). DA-ZP1 efficiently sorted CPEP+ β-like cells by generating cell cultures containing up to 85% CPEP+ β-like cells. A large proportion of purified CPEP+ β-like cells co-expressed PDX1+ and NKX6.1+ (CPEP+PDX1+ cells 85.4% ± 2.6% and CPEP+NKX6.1+ cells 73.6% ± 5.8%). We also detected some glucagon (GCG+) and CPEP+ polyhormonal cells, but most cells were monohormonal (>80% CPEP+GCG−) (Fig 6D). DA-ZP1 positive cells secreted a small but statistically significant amount of insulin in response to high-glucose treatment (1.49 ± 0.08-fold HG versus LG in DA-ZP1+ cells), whereas DA-ZP1 negative cells showed virtually no secretory response (0.99 ± 0.05-fold HG versus LG in DA-ZP1− cells) (Fig 6E). In addition, DA-ZP1+ purified β-like cells responded to both Exendin-4 and KCl stimulation, while DA-ZP1− cells failed to respond to Ex4, but depolarized with KCl treatment (Fig 6E). These data indicate that DA-ZP1 sorting eliminates nonfunctional non-β cells and can be used to isolate β-like cells for functional studies.

### DA-ZP1 is localized to human and mouse pancreatic islet cells

**Figure 7.**
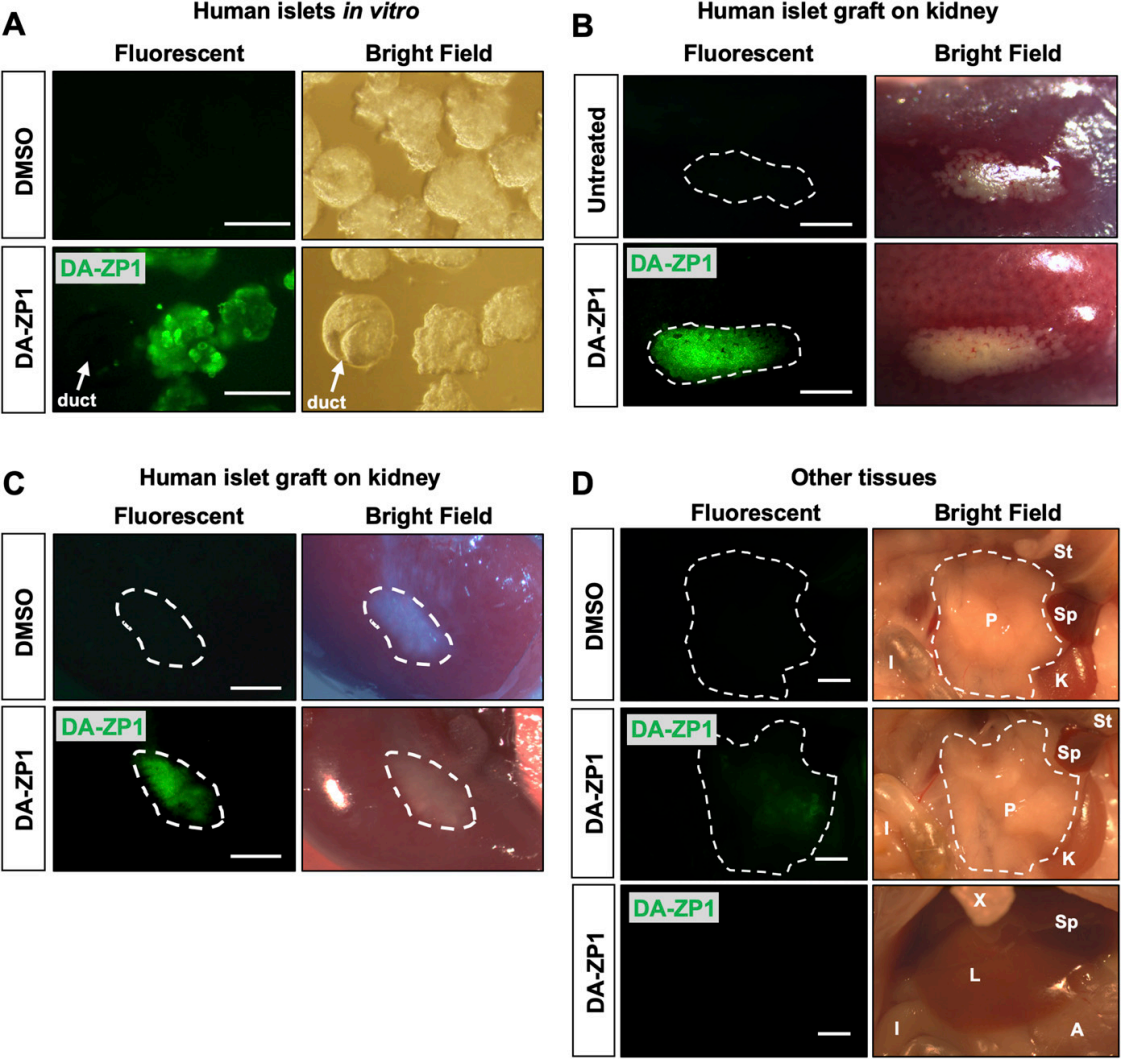
Imaging of human pancreatic islet graft after administration of DA-ZP1. **(A)** Representative images of human pancreatic islets treated with DMSO or 0.8 μM DA-ZP1 for 30 min in vitro. Staining was performed in a 12-well plate (n = 3 replicates) with 25 islets per well. White arrow points to DA-ZP1 negative human pancreatic duct cluster. Scale bar is 200 μm. See also Fig S5. **(B)** Kidneys with grafts were excised 3 d posttransplantation and placed in media with or without DA-ZP1 for 30 min (n = 3 mice/group). No fluorescent signal was detected from untreated graft (top panel); DA-ZP1 treated grafts displayed strong fluorescent signal (bottom panel). The grafts are outlined by white broken lines for better comprehension. Scale bar is 0.15 cm. **(C)** 10 mg/kg BW DA-ZP1 or DMSO was administrated i.v. to mice 1-mo post transplantation (n = 3 mice/group). 1 h after the injection, a strong fluorescent signal was detected in the human islet grafts of DA-ZP1 injected mice, whereas no signal was detected in the grafts of DMSO injected mice. The grafts are outlined by white broken lines for better comprehension. Scale bar 0.15 cm. **(D)** Fluorescent signal was detected only in the pancreas of DA-ZP1 injected mice but not in the DMSO group and no fluorescence signal was detected in any surrounding tissues in both DMSO and DA-ZP1 group (n = 3 mice/group). The pancreas is outlined by white broken lines for better comprehension. St, stomach; P, pancreas; Sp, spleen; K, kidney; I, intestine; X, xiphoid; L, liver; A, adipose. Scale bar 0.15 cm. See also Fig S8.

**Figure S5.**
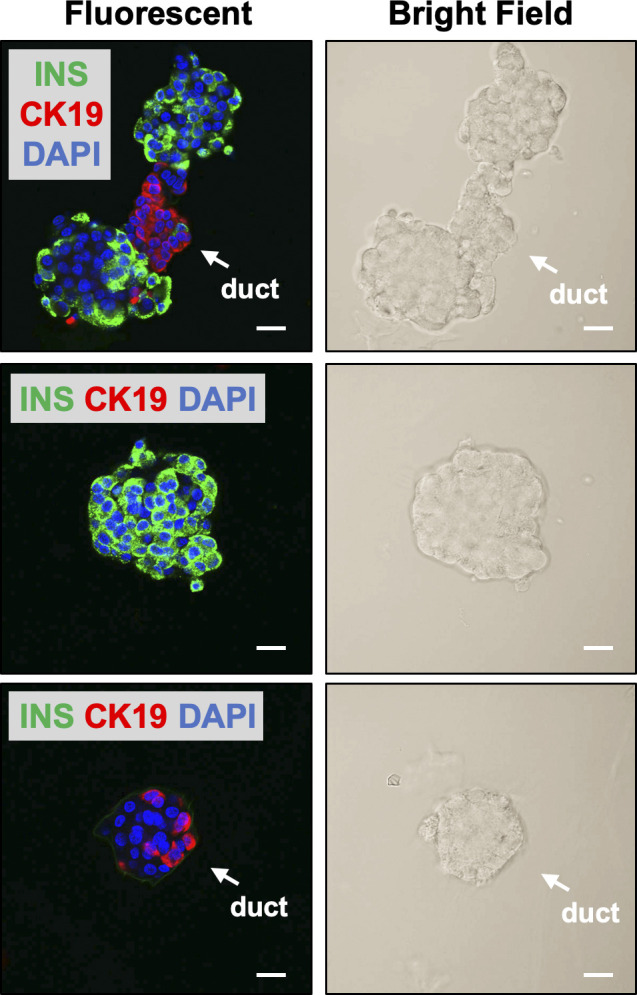
DA-ZP1 is specific to human pancreatic islets. Representative confocal images of pancreatic islets treated with DA-ZP1 and subsequently fixed and stained using antibodies against insulin (green) and CK19 (red) to confirm cell identity. Nuclei stained with DAPI (blue). Scale bar is 20 μm.

**Figure S6.**
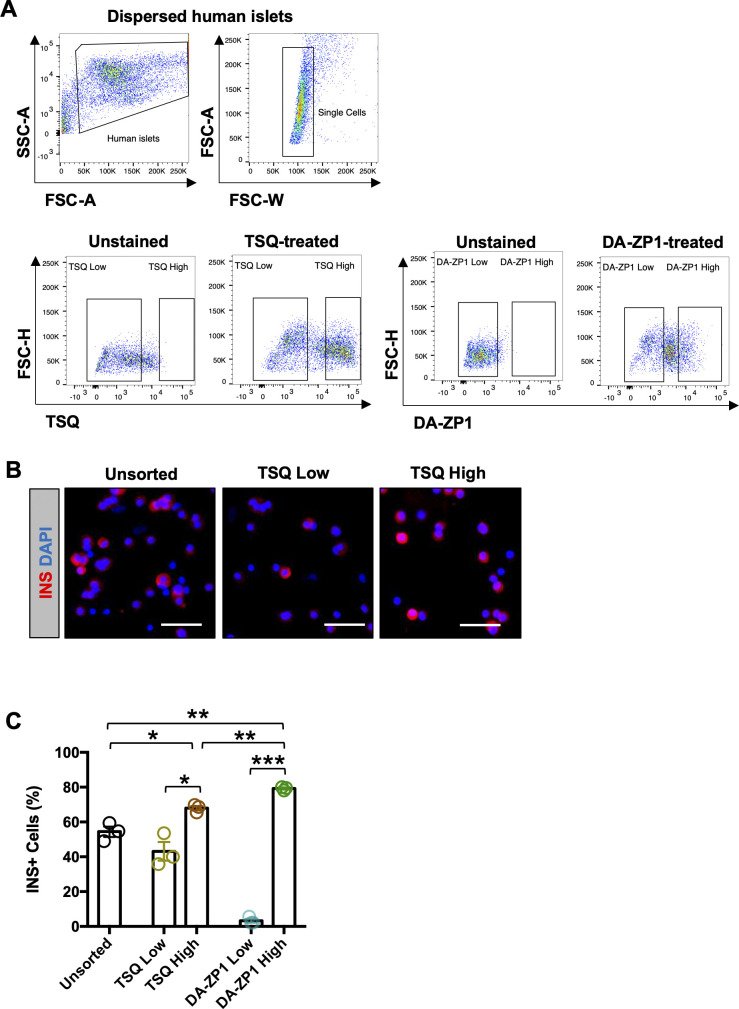
Human β-cell targeting efficiency of DA-ZP1 versus TSQ. **(A)** Representative FACS plots showing gating strategy for sorting human pancreatic islets treated with 1.5 μM TSQ or 0.3 μM DA-ZP1. **(B)** Representative images of dissociated human islet cells treated with TSQ and subsequently fixed and stained for insulin (red). Nuclei stained with DAPI (blue). **(C)** Quantification of dispersed human islets treated with TSQ or DA-ZP1 (n = 3 replicates). Data are represented as mean ± SEM. Multiple *t* test followed by the Holm–Sidak method. *P < 0.05, **P < 0.01, ***P < 0.001.

**Figure S7.**
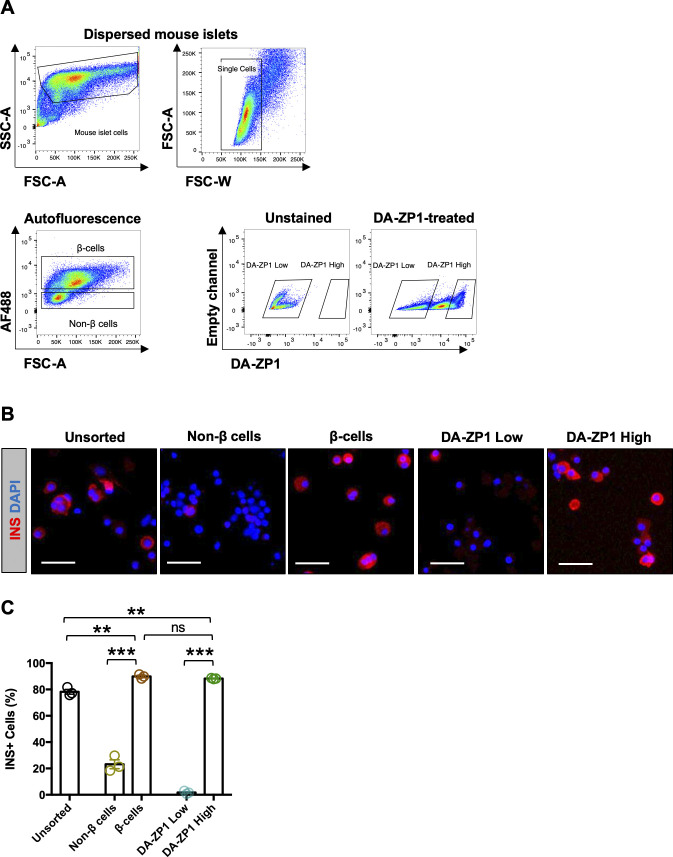
Mouse β-cell targeting efficiency of DA-ZP1 versus autofluorescence. **(A)** Representative FACS plots showing gating strategy for sorting mouse pancreatic islets using autofluorescence-based enrichment or DA-ZP1 treatment. **(B)** Representative images of dissociated mouse islet cells subsequently fixed and stained for insulin (red) after sorting. Nuclei stained with DAPI (blue). **(C)** Quantification of dispersed mouse islets sorted using autofluorescence-based method or after DA-ZP1 treatment (n = 3 replicates). Data are represented as mean ± SEM. Multiple *t* test followed by the Holm–Sidak method. **P < 0.01, **P < 0.001, ns, not significant.

Since DA-ZP1 staining allowed the sorting of live stem-cell derived β-like cells from differentiating cultures, we tested whether it could be used for labeling and tracking human or mouse pancreatic islet cells. Human pancreatic islets were stained with DA-ZP1 in vitro and the fluorescence signal was observed to be restricted to islets and did not label the exocrine tissues (pancreatic ducts) (Figs 7A and S5). We previously demonstrated that ∼81% of the DA-ZP1–targeted pancreatic islet cells were positive for C-peptide (Lee et al, 2020). To compare β-cell targeting efficiency of DA-ZP1 with that of another zinc probe such as TSQ, human islets were treated with TSQ or DA-ZP1 and subsequently sorted (Fig S6A). Immunohistochemical quantification of the sorted islet cells demonstrated that 68% of the TSQ targeted cells and ∼80% of the DA-ZP1 targeted cells were insulin positive (Fig S6B and C), indicating relatively less β-cell targeting efficiency of TSQ. We also demonstrated enrichment of mouse β-cells after DA-ZP1 treatment (∼88% insulin positive cells) that was comparable with the classical granularity/auto-fluorescence-based enrichment process (∼90% insulin positive cells) (Fig S7A–C) (Pipeleers et al, 1985).

**Figure S8.**
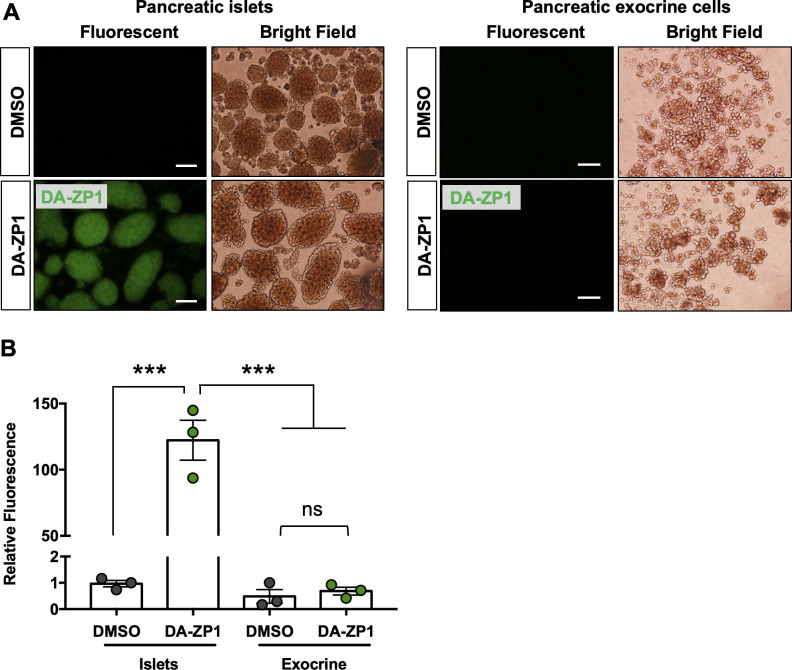
DA-ZP1 is highly specific to pancreatic islet but not exocrine cells. **(A)** Pancreatic islets and pancreatic exocrine cells were isolated 1 h after DMSO or DA-ZP1 injection (n = 3 mice/group). Scale bar is 500 μm. **(B)** Relative fluorescence intensity was measured in the isolated pancreatic islets and exocrine cells after DMSO or DA-ZP1 injection (n = 3 mice/group). Data are represented as mean ± SEM. ***P < 0.001, ns indicates not significant. One-way ANOVA followed by Tukey’s multiple comparison test.

Next, we assessed whether we could use DA-ZP1 to image transplanted human islets in vivo. Human islets were transplanted under the kidney capsule of NSG mice, and kidneys containing the grafts were excised 3 d posttransplantation and treated with DA-ZP1 for 30 min. DA-ZP1–treated grafts were detectable because of their strong fluorescent signal, whereas virtually no fluorescence was detected in the kidney tissue or in the untreated grafts (Fig 7B). To test whether DA-ZP1 can also be used in vivo, mice bearing human islet transplants were housed for a month to allow vascularization of the islet grafts, followed by i.v. injection with 10 mg/kg.b.wt. DA-ZP1 or DMSO. 1 h after the injection, a strong fluorescent signal was detected in the human islet grafts from mice treated with DA-ZP1, whereas no signal was evident in the islet grafts of DMSO-treated animals (Fig 7C). As expected, we also detected fluorescence in the islets of endogenous pancreas of DA-ZP1–injected mice, whereas no signal was observed in the surrounding organs including metabolic tissues such as the liver, adipose tissue, or skeletal muscle in either DA-ZP1− or DMSO-treated animals (Fig 7D). Isolation of pancreatic islets and pancreatic exocrine cells showed that the source of the fluorescence signal in the pancreas was the pancreatic islets, and not exocrine cells (Fig S8A and B). These results indicate that intravenously administered DA-ZP1 specifically reaches the targeted tissue.

## Discussion

The use of fluorescent zinc probes for sorting insulin-positive cells is promising because it satisfies several important criteria: (1) it offers high selectivity for insulin-positive β-cells compared with insulin-negative non-β cells; (2) it is nontoxic to the cells; and (3) it elicits a significant and rapid fluorescent response upon zinc binding, which can be captured by flow cytometry (ex: 495 nm, em: 500–650 nm), minimizing autofluorescence and avoiding UV-induced tissue damage, as might be caused by zinc probes described in earlier reports such as TSQ (Huang & Lippard, 2012).

A recent study showed the importance of endocrine cell clustering for functional maturation of β-like cells in vitro (Nair et al, 2019). They demonstrated that enrichment of GFP+ β-like cells derived from *INS*^GFP/w^ reporter hESC line (∼90%) and their aggregation into cell clusters improved dynamic insulin secretion and calcium signaling in response to secretagogues. In this context the zinc-based DA-ZP1 fluorescence we propose is a very sensitive and reproducible method for purification of β-like cells derived from hESC or iPSC lines and could be applied to generate highly pure β-like cell clusters for improved functional maturation.

Until now, antibody-based methods have been used in an attempt to enrich β-cells from islets. For example, in efforts to study individual human islet cell types, a panel of cell–surface markers was developed (Dorrell et al, 2008, 2016) wherein live human pancreatic β-cells were isolated by depleting other cell types. More recently, NTPDase3 was reported to be a cell–surface biomarker and used to purify human β-cells in combination with negative selection markers (Saunders et al, 2019). To our knowledge, NTPDase3 has not been tested using its cell surface marker properties to show whether it enables isolation of β-like cells. In another study, CD49a, which is not specific to human β-cells, was reported as a surface marker of stem cell-derived β-like cells (Veres et al, 2019). Anti-CD49a labeling followed by magnetic sorting was used to purify β-like cells up to 80%. Compared with these antibody selection approaches, DA-ZP1 is very rapid, inexpensive, and can be applied to sort insulin-positive β-like cells without the need for cell surface marker expression. Isolation of a viable β-like population with robust enrichment should enable transcriptional studies and functional assays such as static glucose stimulation and other metabolic analyses.

Our data confirm the selective presence of the zinc-reaction probe DA-ZP1 in pancreatic islets in vivo and demonstrate the potential utility of the system as a tool for β-cell imaging. Development of near infrared (NIR)–emitting zinc-selective sensors could permit visualization of tissues that are located anatomically deep from the surface effectively, and could enable noninvasive imaging of dynamic changes in β-cell mass (Hilderbrand & Weissleder, 2010; Smith et al, 2010). Therefore, future studies are warranted to evaluate if NIR zinc-selective sensors can be used to noninvasively monitor changes in the mass of transplanted human islets or endogenous β-cells. Zinc-reaction probes could also be developed for applications such as β-cell-targeted delivery (Horton et al, 2019; Lee et al, 2020). For example, drugs with an appended zinc-reaction probe could be delivered selectively to pancreatic β-cells to enhance function, and promote survival, or proliferation of target cells. Zn(II)-mediated hydrolytic cleavage is able to release the drug from zinc-reaction probes and switch the inactive drug to an active compound. The ability of the Zn(II)-mediated switch to deliver active compounds warrants in vivo experiments to evaluate the safety and efficacy of drug-attached zinc-reaction probes as a β-cell-specific delivery method.

Finally, the zinc-based approach can be applied to improve the yield of pure β-like cells in differentiation approaches currently being used. Although several protocols can generate functional β-like cells by stepwise differentiation of hESC lines or patient-derived iPSC lines, they also include non-β cells likely because of variable differentiation efficiency (Thatava et al, 2013; Nishizawa et al, 2016). The use of zinc-binding fluorescence probes allows enrichment of β-like cells containing insulin granules and holds an advantage over the cell surface antibody approach that does not necessarily enrich for functional insulin-positive cells.

In sum, the use of zinc-binding fluorescence probes such as DA-ZP1 provides a unique opportunity for enrichment and purification of insulin-positive β-like cells, for a better understanding of human β-cell development, investigation of disease mechanisms (in vitro disease modeling), and for drug discovery.

## Materials and Methods

### Cell culture

All cells (cell lines and primary cells) were cultured at 37°C, 5% CO_2_, and 95% humidity and manipulated in a sterile laminar flow hood. Human embryonic kidney 293 cells were cultured in DMEM High Glucose media supplemented with 10% FBS and maintained at low passage number <10. The EndoC-βH1 cells were cultured and passaged as previously described (Ravassard et al, 2011). Briefly, the culture flask was coated with DMEM HG (glucose 4.5 g/l) containing penicillin–streptomycin (1%), fibronectin (2 μg/ml), and ECM (1% vol/vol) and incubated for at least 1 h in 5% CO_2_ at 37°C before the cells were seeded. EndoC-βH1 cells were grown on ECM/fibronectin coated culture flasks containing DMEM LG (glucose 1 g/l), BSA fraction V (2% wt/vol), β-mercaptoethanol (50 μM), nicotinamide (10 mM), transferrin (5.5 μg/ml), sodium selenite (6.7 ng/ml), and penicillin–streptomycin (1%).

### hPSC culture and in vitro differentiation

The hESC lines, H1 and H9, were grown on vitronectin (VTN-N; Thermo Fisher Scientific) coated dishes using chemically defined Essential 8 medium (E8; Thermo Fisher Scientific) and regularly confirmed to be mycoplasma-free by MycoAlert Mycoplasma Test Kit (Lonza). The medium was changed every day, and cells were passaged every 5–6 d using 0.5 mM EDTA. The iPSC lines iAG16102, iN805-6, and iN65-51 were derived from human fibroblasts and have been reported previously (Teo et al, 2013). Cells were grown on VTN-N–coated dishes using E8 medium and regularly confirmed to be mycoplasma-free. The medium was changed every day, and cells were passaged every 5–6 d using 0.5 mM EDTA. hESCs or iPSCs were differentiated towards β-like stage (Stage 6) using previously published protocols with some modifications (Pagliuca et al, 2014; Rezania et al, 2014). Briefly, hESCs or iPSCs were dissociated into single cells using TrypLE (Thermo Fisher Scientific) and plated on VTN-N coated plates in E8 medium supplemented with 10 μM Rho-associated protein kinase (ROCK) inhibitor Y-27632 (Chemdea) at >90% confluency 1 d before initiation of differentiation. Cultures were rinsed with DPBS without Mg^2+^ and Ca^2+^ (Gibco) and differentiation medium was added. Differentiation medium used for each stage is given in Table S1.

Table S1 Composition of human pluripotent stem cell differentiation media.

### Human islet processing

Human islets were obtained from the Integrated Islet Distribution Program. Human islets were processed for DA-ZP1 treatment or transplantation. Joslin Diabetes Center Institutional Review Board declared studies on de-identified autopsy tissue does not qualify as human subject research. Upon receipt, islets were centrifuged at 200*g* for 1 min and resuspended in Miami media (Mediatech). Islets were then transferred to 10 cm culture dishes and cultured overnight to 24 h. Healthy islets were handpicked and washed with DPBS. For transplantation experiments 500 IEQ were transplanted under the kidney capsule of NSG (NOD-*scid*-IL2Rg^null^; Jackson Laboratory) mice as previously described (Kahraman et al, 2011). For DA-ZP1 staining, islets were incubated in culture medium containing DA-ZP1 for 30 min at 37°C. At the end of incubation time, cells were washed and cell pellet was resuspended in DA-ZP1 free Miami media. Donor demographic information is summarized in Table S2.

Table S2 Donor information.

### Immunostaining

Dissociated islet cells were immediately seeded after sorting in matrigel (Corning) coated flat bottom 96 well plates and fixed next day. Cells growing in culture dishes were fixed in 4% PFA (Wako) for 15 min at room temperature and washed with PBS three times. For confocal imaging, EndoC-βH1 cells were fixed in cold methanol to preserve fluorescence (Jamur & Oliver, 2010). Cells were then permeabilized with PBS containing 0.25% Triton-X for 30 min at room temperature and blocked with PBS containing 0.25% Triton-X and 5% donkey serum (Sigma-Aldrich) for 1 h at room temperature. Primary antibody was diluted in antibody dilution buffer (Dako) and added to the wells for overnight incubation at 4°C. Cells were washed three times with PBS and the secondary antibody, diluted in PBS, was added to the wells for 1 h at room temperature. Cells were washed three times with PBS and DAPI (Sigma-Aldrich) was added to the wells. Images were captured using an Olympus IX51 Inverted Microscope and cellSens Standard Software Zeiss LSM 710 NLO confocal laser scanning microscope. Pancreatic islets and ductal clusters were fixed in 10% formalin and then stained using the whole-mount immunostaining method as described by Rezanejad et al (2019). Antibody information is given in Table S3.

Table S3 Antibody information.

### In vitro imaging assays

Staining was conducted in 96-well format. DA-ZP1 or DM-1 was dissolved in DMSO and added to the DMEM cell medium without phenol red (Thermo Fisher Scientific) at final concentration. The culture medium was then replaced with compound-containing medium for 30 min at 37°C. At the end of incubation time, cells were washed with DPBS and fresh cell medium containing DAPI was added to the wells. The cells were imaged immediately or fixed in 4% PFA for 15 min at room temperature and washed with PBS for imaging later. Images were acquired using an automated system (n = 9 images/well) and analyzed by using MetaXpress software. For the Figs 1 and S8A, fluorescent images were taken by Olympus IX51 Inverted Microscope and relative intracellular fluorescence was measured by Image J. Briefly, ∼40 cells were randomly selected and integrated density was measured for each cell (4 samples/group). Background fluorescence was subtracted to calculate corrected total cell fluorescence. For the Fig S8A, 15 islets or exocrine clusters were randomly selected for each group and the mean fluorescent intensity was measured (n = 3 mice/group).

### DA-ZP1 treatment and FACS

Cells were harvested using trypsin and neutralized in DMEM containing 10% FBS. Cell pellet was resuspended in DA-ZP1 containing cell media and incubated in 37°C for 30 min. At the end of incubation time, cells were washed with DPBS and cell pellet resuspended in fresh DA-ZP1–free media. Cells were analyzed and sorted by Aria (Joslin Flow Cytometry Core). Increase in sorting time does not alter staining patterns because fluorescence response is maintained by cells after treatment with DA-ZP1. For TSQ staining, cell pellet was resuspended in TSQ (Enzo) containing FACS Buffer (2% FBS in PBS) and cells were exposed to TSQ during sorting. For cell tracking experiments (Fig S2), EndoC-βH1 and 293 cells were labeled with either the Violet or Far Red fluorescent dye (Thermo Fisher Scientific), respectively. Briefly, five million cells were harvested, pelleted, resuspended in 5 ml staining solution (1 μM fluorescent dye in DPBS), and incubated in 37°C for 20 min. Fluorescently labeled cells were washed with 25 ml DMEM 10% FBS and incubated in 37°C for 5 min. Cells were spun down, stained with Zombie Red viability dye (BioLegend) followed by DA-ZP1 staining, fixed and analyzed by flow cytometry (BD LSRFortessa High Throughput Sampler; BD Biosciences, Joslin Flow Cytometry Core). Analysis of flow cytometry data was completed using FlowJo 10.4.2 (FlowJo LLC). Gating strategy is shown in Fig S2.

### FACS

For Ki67 staining, EndoC-βH1 cells were treated with DA-ZP1 (0, 0.16, 1.25, 10 μM) for 1 h and cultured in DA-ZP1–free media for 24 h. Cells were trypsinized, washed with DPBS, and stained with Zombie NIR Viability dye (BioLegend) and then fixed in 4% PFA for 15 min at room temperature. Cells were then spun and washed with cold FACS buffer (5% FBS in PBS). Permeabilization and blocking were carried out on ice for 1 h in PBS containing 5% donkey serum and 0.2% TritonX. Antibody staining was performed 1 h at 4°C. Antibody information is given in Table S3. For CPEP staining, cells were harvested and fixed in 4% PFA for 15 min at room temperature. Cells were then spun and washed with cold FACS buffer (5% FBS in PBS). Permeabilization and blocking was carried out on ice for 1 h in PBS containing 5% donkey serum and 0.2% TritonX. Antibody staining was performed overnight at 4°C followed by incubation with secondary antibody for 1 h on ice. Cells were washed with FACS buffer, resuspended, and filtered through a 30-μm filter before analysis by LSRII (BD Biosciences, Joslin Flow Cytometry Core). Gating was determined according to the secondary-only or isotype controls.

### Apoptosis detection

Cells were trypsinized, washed with DPBS, and stained with Zombie NIR Viability dye (BioLegend) for 15 min at RT. Cells were washed first with medium containing 10% FBS, followed by a wash with 1× binding buffer, and resuspended in 1× binding buffer containing APC Annexin V (1:20; BD Biosciences). Cells were incubated for 15 min at RT and analyzed by FACS LSRII (Joslin Flow Cytometry Core). Apoptotic cell rate was determined as percentage of Annexin V+ and Zombie NIR− cells.

### Ex vivo imaging

NSG adult male mice were obtained from Jackson Laboratories at 8–12 wk of age for human islet transplantation studies. Mice were maintained at Joslin Animal Facility on a 12:12 h light:dark cycle with ad lib access to water and standard rodent chow. All procedures were approved by the Joslin Diabetes Center Institutional Animal Care and Use Committee and performed in accordance with National Institutes of Health (NIH) guidelines. Human islet transplanted kidneys were excised 3 d posttransplantation and placed in Miami media containing 0 or 3 μM DA-ZP1 and incubated in 37°C water bath for 30 min. Human islet grafts were imaged using SteREo Discovery V8 dissection microscope equipped with 0.63× objective, X-Cite series 120Q light source, and Axiocam 512 color camera. Images were analyzed by using Zen 2.3 lite software.

10 mg/kg BW DA-ZP1 was prepared in 125 μl saline solution and injected via tail vein into mice bearing human islet grafts 1-mo posttransplantation. 1 h after the injection, mice were anesthetized by ketamine 100 mg/kg/xylazine 10 mg/kg injection (i.p.). The kidney with the graft was exposed through an incision and imaged using SteREo Discovery V8 dissection microscope equipped with Axiocam 512 color camera. Abdominal V-incision was made to image liver, pancreas, and surrounding tissues.

### Pancreatic islet and exocrine tissue isolation

Pancreatic islets and exocrine cells were obtained from 8-wk-old NSG mice 1 h after DA-ZP1 or DMSO injection. Mice were anesthetized by i.p. ketamine/xylazine injection and 2.6 ml CIzyme RI solution (30k CDA U, resuspended in RPMI 1640; VitaCyte) was injected through the pancreatic duct using 27 g-needle. Inflated pancreas was transferred to a 50 ml tube for digestion in 37°C water bath for 17 min. The tubes were hand-shaken vigorously for 5–10 s and washed twice with ice-cold RPMI containing 10% FBS. Tissue was filtered through a 424 μm sieve to remove undigested tissue and fat. The islets and exocrine cells were separated using a Histopaque 1077 (Sigma-Aldrich) density gradient. The islets were collected from the top interface and the exocrine cells were collected from the pellet and transferred to new 50 ml tube. After three washes with RPMI 1640 medium containing 10% FBS, purified islets and exocrine cells were handpicked under the dissection microscope and transferred to a 12 well plates. Cells were stained with Propidium Iodide (1 μg/ml; Sigma-Aldrich) to determine cell viability. Fluorescent and bright field images of live cells were taken immediately by Olympus IX51 Inverted Microscope.

### Insulin secretion assay

EndoC-βH1 cells were starved overnight in 2.8 mM glucose followed by 1 h incubation in Krebs Ringer Buffer (KRB) containing NaCl (115 mM), NaHCO_3_ (24 mM), KCl (5 mM), MgCl_2_ (1 mM), CaCl_2_ (1 mM), Hepes (10 mM), BSA (0.2% wt/vol), and 0.5 mM glucose. Static insulin secretion assays were then initiated by adding KRB containing 3.3 or 16.7 mM glucose for 1 h. Aliquots of supernatants were removed for later analysis and ice-cold acid ethanol was added to extract insulin content from cells. Insulin secretion and content were measured by the human insulin ELISA (Mercodia) according to the manufacturer’s instructions.

β-like cells were starved 1 h in KRB containing 0.5 mM glucose. Insulin secretion was stimulated by adding KRB containing 1 mM glucose (LG), 20 mM glucose (HG), and 10 nM Exendin-4 (Sigma-Aldrich) in the presence of 20 mM glucose or 30 mM KCl for 1 h. Supernatant samples were collected, cell debris were removed by centrifugation, and insulin levels were measured. Human insulin secretion was calculated by dividing the secreted insulin by the total number of cells.

### Statistical analysis

Statistical analysis was performed by *t* test or ANOVA. All values are ± SEM, and statistical significance was set at *P* < 0.05.

## Data Availability

The data that support the findings of this study are available from the corresponding author upon reasonable request.

## Supplementary Material

Reviewer comments
