# Supplementary material for: Harnessing reaction-based probes to preferentially target pancreatic β-cells and β-like cells
Source: Life Sci Alliance. 2021 Jan 28;4(4):e202000840. doi: 10.26508/lsa.202000840 (PMC7898467; doi:10.26508/lsa.202000840)
Supplement: Reviewer comments [file LSA-2020-00840_review_history.pdf]

Life Science Alliance

Harnessing Reaction-Based Probes to Preferentially Target Pancreatic b-Cells and b-Like Cells

Sevim Kahraman, Debasish Manna, Ercument Dirice, Basudeb Maji, Jonnell Small, Bridget Wagner, Amit Choudhary, and Rohit Kulkarni
DOI: https://doi.org/10.26508/lsa.202000840

Corresponding author(s): Rohit Kulkarni, Joslin Diabetes Center and Amit Choudhary, Broad Institute of MIT and Harvard

Review Timeline:

Submission Date:	2020-07-05
Editorial Decision:	2020-08-10
Revision Received:	2020-12-02
Editorial Decision:	2021-01-04
Revision Received:	2021-01-07
Editorial Decision:	2021-01-08
Revision Received:	2021-01-11
Accepted:	2021-01-12

Scientific Editor: Shachi Bhatt

Transaction Report:

(Note: With the exception of the correction of typographical or spelling errors that could be a source of ambiguity, letters and reports are not edited. The original formatting of letters and referee reports may not be reflected in this compilation.)

No full Peer Review Process File is available with this article, as the authors have chosen not to make the review process public in this case.

August 10, 2020

Re: Life Science Alliance manuscript #LSA-2020-00840-T

Prof. Rohit N. Kulkarni
Joslin Diabetes Center
Islet Cell and Regenerative Biology
One Joslin Place
Boston, MA 2215

Dear Dr. Kulkarni,

Thank you for submitting your manuscript entitled "Harnessing Reaction-Based Probes to Preferentially Target Pancreatic b Cells and b-like Cells" to Life Science Alliance.

The manuscript was assessed by expert reviewers, (comments are appended to this letter) and as you can see, the reviewers were quite enthusiastic about the study, but have pointed out some concerns that should be addressed before the manuscript can be published at Life Science Alliance. We encourage you to submit a revised version of this study that addresses all of the concerns pointed out by Rev 1 and 2, and Rev 3 pt 3. While we appreciate the detailed review by Rev 3, the experimental data in response to Rev 3, pt 1, 2 and 4 should only be included in the revised manuscript if it is readily available or readily attainable, if not a detailed discussion regarding these concerns should be included in the revised manuscript.

To upload the revised version of your manuscript, please log in to your account:
https://lsa.msubmit.net/cgi-bin/main.plex
You will be guided to complete the submission of your revised manuscript and to fill in all necessary information. Please get in touch in case you do not know or remember your login name.

We would be happy to discuss the individual revision points further with you should this be helpful.

While you are revising your manuscript, please also attend to the below editorial points to help expedite the publication of your manuscript. Please direct any editorial questions to the journal office.

The typical timeframe for revisions is three months. Please note that papers are generally considered through only one revision cycle, so strong support from the referees on the revised version is needed for acceptance.

When submitting the revision, please include a letter addressing the reviewers' comments point by point.

We hope that the comments below will prove constructive as your work progresses.

Thank you for this interesting contribution to Life Science Alliance. We are looking forward to receiving your revised manuscript.

Sincerely,

Reilly Lorenz
Editorial Office Life Science Alliance
Meyerhofstr. 1
69117 Heidelberg, Germany
t +49 6221 8891 414
e contact@life-science-alliance.org
www.life-science-alliance.org

A. THESE ITEMS ARE REQUIRED FOR REVISIONS

-- A letter addressing the reviewers' comments point by point.

-- An editable version of the final text (.DOC or .DOCX) is needed for copyediting (no PDFs).

-- High-resolution figure, supplementary figure and video files uploaded as individual files: See our detailed guidelines for preparing your production-ready images, http://www.life-science-alliance.org/authors

-- Summary blurb (enter in submission system): A short text summarizing in a single sentence the study (max. 200 characters including spaces). This text is used in conjunction with the titles of papers, hence should be informative and complementary to the title and running title. It should describe the context and significance of the findings for a general readership; it should be written in the present tense and refer to the work in the third person. Author names should not be mentioned.

B. MANUSCRIPT ORGANIZATION AND FORMATTING:

Full guidelines are available on our Instructions for Authors page, http://www.life-science-alliance.org/authors

We encourage our authors to provide original source data, particularly uncropped/-processed electrophoretic blots and spreadsheets for the main figures of the manuscript. If you would like to add source data, we would welcome one PDF/Excel-file per figure for this information. These files will be linked online as supplementary "Source Data" files.

IMPORTANT: It is Life Science Alliance policy that if requested, original data images must be made available. Failure to provide original images upon request will result in unavoidable delays in publication. Please ensure that you have access to all original microscopy and blot data images before submitting your revision.

January 4, 2021

RE: Life Science Alliance Manuscript #LSA-2020-00840-TR

Prof. Rohit N. Kulkarni
Joslin Diabetes Center
Islet Cell and Regenerative Biology
One Joslin Place
Boston, MA 2215

Dear Dr. Kulkarni,

Thank you for submitting your revised manuscript entitled "Harnessing Reaction-Based Probes to Preferentially Target Pancreatic b-Cells and b-like Cells". We would be happy to publish your paper in Life Science Alliance pending final revisions necessary to meet our formatting guidelines.

Along with the points listed below, please also attend to the following,
-please consult our Manuscript Preparation Guidelines https://www.life-science-alliance.org/manuscript-prep and put your manuscript sections in the correct order
-please add ORCID ID for corresponding and secondary corresponding author-you should have received instructions on how to do so

If you are planning a press release on your work, please inform us immediately to allow informing our production team and scheduling a release date.

To upload the final version of your manuscript, please log in to your account: https://lsa.msubmit.net/cgi-bin/main.plex
You will be guided to complete the submission of your revised manuscript and to fill in all necessary information. Please get in touch in case you do not know or remember your login name.

To avoid unnecessary delays in the acceptance and publication of your paper, please read the following information carefully.

A. FINAL FILES:

These items are required for acceptance.

-- An editable version of the final text (.DOC or .DOCX) is needed for copyediting (no PDFs).

-- High-resolution figure, supplementary figure and video files uploaded as individual files: See our detailed guidelines for preparing your production-ready images, https://www.life-science-alliance.org/authors

-- Summary blurb (enter in submission system): A short text summarizing in a single sentence the study (max. 200 characters including spaces). This text is used in conjunction with the titles of papers, hence should be informative and complementary to the title. It should describe the context and significance of the findings for a general readership; it should be written in the present tense

and refer to the work in the third person. Author names should not be mentioned.

B. MANUSCRIPT ORGANIZATION AND FORMATTING:

Full guidelines are available on our Instructions for Authors page, https://www.life-science-alliance.org/authors

We encourage our authors to provide original source data, particularly uncropped/-processed electrophoretic blots and spreadsheets for the main figures of the manuscript. If you would like to add source data, we would welcome one PDF/Excel-file per figure for this information. These files will be linked online as supplementary "Source Data" files.

Submission of a paper that does not conform to Life Science Alliance guidelines will delay the acceptance of your manuscript.

It is Life Science Alliance policy that if requested, original data images must be made available to the editors. Failure to provide original images upon request will result in unavoidable delays in publication. Please ensure that you have access to all original data images prior to final submission.

The license to publish form must be signed before your manuscript can be sent to production. A link to the electronic license to publish form will be sent to the corresponding author only. Please take a moment to check your funder requirements.

Reviews, decision letters, and point-by-point responses associated with peer-review at Life Science Alliance will be published online, alongside the manuscript. If you do want to opt out of having the reviewer reports and your point-by-point responses displayed, please let us know immediately.

Thank you for your attention to these final processing requirements. Please revise and format the manuscript and upload materials within 7 days.

Thank you for this interesting contribution, we look forward to publishing your paper in Life Science Alliance.

Sincerely,

Shachi Bhatt, Ph.D.
Executive Editor
Life Science Alliance
https://www.lsajournal.org/
Tweet @SciBhatt @LSAjournal

January 8, 2021

RE: Life Science Alliance Manuscript #LSA-2020-00840-TRR

Prof. Rohit N. Kulkarni
Joslin Diabetes Center
Islet Cell and Regenerative Biology
Room 410
One Joslin Place
Boston, MA 2215

Dear Dr. Kulkarni,

Thank you for submitting your revised manuscript entitled "Harnessing Reaction-Based Probes to Preferentially Target Pancreatic b-Cells and b-Like Cells". As we were QC'ing the files for acceptance, we noticed that there are 2 things still missing,

+ please separate the Results and Discussion section into two - 1. Results 2. Discussion, as per our formatting requirements
+ please provide the ORCID Id for the second corresponding author. They should have received instructions in their email as to how to update it on their profile.

To upload the final version of your manuscript, please log in to your account:
https://lsa.msubmit.net/cgi-bin/main.plex
You will be guided to complete the submission of your revised manuscript and to fill in all necessary information. Please get in touch in case you do not know or remember your login name.

Submission of a paper that does not conform to Life Science Alliance guidelines will delay the acceptance of your manuscript.

Reviews, decision letters, and point-by-point responses associated with peer-review at Life Science Alliance will be published online, alongside the manuscript. If you do want to opt out of having the reviewer reports and your point-by-point responses displayed, please let us know immediately.

Thank you for your attention to these final processing requirements. Please revise and format the manuscript and upload materials within 7 days.

Thank you for this interesting contribution, we look forward to publishing your paper in Life Science Alliance.

Sincerely,

Shachi Bhatt, Ph.D.
Executive Editor
Life Science Alliance
https://www.lsajournal.org/

Tweet @SciBhatt @LSAjournal

January 12, 2021

RE: Life Science Alliance Manuscript #LSA-2020-00840-TRRR

Prof. Rohit N. Kulkarni
Joslin Diabetes Center
Islet Cell and Regenerative Biology
Room 410
One Joslin Place
Boston, MA 2215

Dear Dr. Kulkarni,

Thank you for submitting your Research Article entitled "Harnessing Reaction-Based Probes to Preferentially Target Pancreatic b-Cells and b-Like Cells". It is a pleasure to let you know that your manuscript is now accepted for publication in Life Science Alliance. Congratulations on this interesting work.

The final published version of your manuscript will be deposited by us to PubMed Central upon online publication.

Your manuscript will now progress through copyediting and proofing. It is journal policy that authors provide original data upon request.

Reviews, decision letters, and point-by-point responses associated with peer-review at Life Science Alliance will be published online, alongside the manuscript. If you do want to opt out of having the reviewer reports and your point-by-point responses displayed, please let us know immediately.

IMPORTANT: If you will be unreachable at any time, please provide us with the email address of an alternate author. Failure to respond to routine queries may lead to unavoidable delays in publication.

Scheduling details will be available from our production department. You will receive proofs shortly before the publication date. Only essential corrections can be made at the proof stage so if there are any minor final changes you wish to make to the manuscript, please let the journal office know now.

DISTRIBUTION OF MATERIALS:
Authors are required to distribute freely any materials used in experiments published in Life Science Alliance. Authors are encouraged to deposit materials used in their studies to the appropriate repositories for distribution to researchers.

You can contact the journal office with any questions, contact@life-science-alliance.org

Again, congratulations on a very nice paper. I hope you found the review process to be constructive and are pleased with how the manuscript was handled editorially. We look forward to future exciting submissions from your lab.

Sincerely,

Shachi Bhatt, Ph.D.
Executive Editor
Life Science Alliance
https://www.lsajournal.org/
Tweet @SciBhatt @LSAjournal
